# Effect of Dietary Blue-Green Microalgae Inclusion as a Replacement to Soybean Meal on Laying Hens’ Performance, Egg Quality, Plasma Metabolites, and Hematology

**DOI:** 10.3390/ani12202816

**Published:** 2022-10-18

**Authors:** Ahmed O. Abbas, Abdulaziz A. Alaqil, Gamal M. K. Mehaisen, Nancy N. Kamel

**Affiliations:** 1 Department of Animal and Fish Production, College of Agricultural and Food Sciences, King Faisal University, P.O. Box 420, Al-Ahsa 31982, Saudi Arabia; 2 Department of Animal Production, Faculty of Agriculture, Cairo University, 7 Gamma St., Giza 12613, Egypt; 3 Department of Animal Production, National Research Centre, El Buhouth St., Dokki, Giza 12622, Egypt

**Keywords:** *Spirulina platensisis*, laying hens, performance, egg traits, metabolites, hematological parameters

## Abstract

**Simple Summary:**

In recent years, a variety of natural products and medicinal plants have been widely implemented as alternative nutritional strategies for poultry production. This study aimed to investigate the possible impact of different dietary levels of spirulina microalgae on the productive performance, egg traits, blood metabolites, and hematological parameters of laying hens. Based on the findings, the spirulina could be contained within the layer diets as an alternative source of protein, with positive impacts on the laying hens’ production and physiological performance.

**Abstract:**

*Spirulina platensisis* (SP) is a blue-green microalgae with a high value for animal and poultry nutrition. The study employed 250 40-week-old, HY-Line W-36 commercial laying hens. The layers received one of five experimental diet substitutes in five groups for 10 consecutive weeks (five replicates of 10 hens each group); a soybean-corn basal diet formulation without SP (Control group) or the soybean partially substituted with 3% SP, 6% SP, 9% SP, and 12% SP (for the remaining four groups). The results showed that dietary SP treatment significantly (*p* < 0.05) improved the productive performance, egg quality, blood metabolites, and hematological parameters of laying hens. In addition, there were linear and quadratic effects for increasing the levels of SP inclusion into the layer diets; however, the highest values of most parameters were observed when using 9% SP (90 g/kg of the layer diets). Furthermore, the results showed that 4.7% of the soybean meal ingredient in the layer diet could be replaced by 1% of SP. In conclusion, the partial replacement of soybean meal by SP into layer diets could be used as a promising nutritional approach to optimize the performance of laying hens.

## 1. Introduction

SP is a blue-green microalgae which grows naturally in brackish water and lakes in several countries [1]. It gained prominence since it was used as a source of food during long-term space missions [2]. SP has abundant contents of the macro- and micronutrients, and therefore, it is used to produce safe nutritional and medicinal compounds [3,4]. It could be simply harvested and commercially processed into appropriate forms to be used as human and animal nutrition [5,6,7,8,9]. SP comprises high levels of protein (50–70%), carbohydrates (15–20%), lipids (5–7%), minerals (6–9%), and vitamins (1–4%) [1,10]. SP provides most of the essential amino acids, fatty acids, carotenoids, and other phytopigments [1]. It is a unique source of vitamin B12, γ-linolenic fatty acid, β-carotene, calcium, and iron [10].

The nutritional value of SP has attracted many researchers to study the effectiveness and the functional mechanism of the spirulina on animal species. It has been reported that SP enhances the productive performance and improves the general health of different animals [11,12,13,14]. In addition, it has been displayed that SP has favorable biological properties such as immunomodulation [15,16], tumor resistance [17], anti-inflammation [18], lowering-cholesterol [19], and antioxidant activation [20].

In particular, dietary supplementation with spirulina has positive results in poultry species. It was demonstrated that traditional protein sources of broiler diets could be substituted with up to 15% SP without negatively affecting the meat quality and production [21,22,23]. Additionally, it was reported that inclusion of 0.25–1.0% SP into broiler diets improved the growth efficiency, the nutrients digestibility, the antioxidant enzyme activity, the anti-inflammatory response, and the cecal microflora population [14,24,25]. In addition, Feshanghchi et al. [26] found that supplementing SP at 1% to broiler diets can effectively mitigate the negative effects of aflatoxins on the broiler performance, immunological function, and blood biochemical characteristics. In recent studies, it was found that adding 0.5–2% SP to broiler and quail diets can relieve the deleterious results of heat stress on the growth, carcass, redox, and physiological characteristics [12,27,28,29]. In contrast, feeding 2% SP to laying hens enhanced their egg yolk color and shell thickness and strength, and improved their liver functions, egg lipid profile, and antioxidant status [30,31]. Other studies [13,32,33] displayed that supplementing SP at 0.2–2.5% into the laying hen rations enhanced only the egg yolk color without exhibiting any effect on the other egg parameters.

According to our knowledge, most of the works of the literature investigating the dietary SP supplementation in laying hens applied no more than 5% SP as a feed additive into their basal diets [34,35]. In addition, there is a lack of sufficient studies discussing the possible effect of SP administration as a main diet ingredient replacing the traditional sources of protein into layer diets. The goal of this study was to highlight the potential impact of inserting various doses of SP as a replacement to soybean meal into laying hens’ diets on their performance, eggs traits, metabolites, and hematology profile.

## 2. Materials and Methods

### 2.1. Birds and SP Treatments

Two hundred and fifty 40-week-old, HY-Line W-36 commercial laying hens were placed individually in 40 × 40 × 50 cm cages maintained in an open housing system. The hens were kept in standard environmental and hygienic conditions. The hens were provided with a daily lighting system (16L/8D), *ad libitum* feed, and unlimited access to water. The hens were randomly allocated into five equal groups (5 replicates × 10 hens each, per group) and fed on one of five experimental diets for 10 consecutive weeks (40–50 weeks of age). The diets were formulated as mash to meet the nutritional recommendations for HY-Line W-36 layers (Table 1). The first group received a soybean–corn basal diet formulation without SP supplementation (Control group). The other four groups received a basal soybean–corn formulation diet substituted with 3%, 6%, 9%, and 12% *Spirulina platensis* microalgae (3% SP, 6% SP, 9% SP, and 12% SP groups, respectively). The SP microalgae was obtained as a freeze-dried powder form (Inner Mongolia Rejuve Biotech. Co., Ltd., Ordos, China) and mixed daily into the diet of the laying hens. The rest of the feed was sieved to check the percentage of the residual SP as an indicator for consuming SP by the birds without separation from the diet. The minimum requirements for poultry nutrition [36] were considered to figure out the nutrients of the experimental diets. The minerals and amino acid profile of soybean meal (SBM) and SP are presented in Table 1, according to the pamphlet of the commercial supplier. The nutritional analysis of the SP and the experimental diets were carried out using the guidelines of the Association of Official Analysis Chemists [37], and are included in Table 1 and Table 2, respectively.

### 2.2. Productive Performance

The hens were weighed at 40 and 50 wk of age to determine the initial and final body weights (IBW and FBW, respectively). Eggs were collected and weighed daily to assess the average hen daily egg production (EP%) and egg weight (EW) throughout the experiment. The feed intake (FI) was recorded daily. Feed conversion ratio (FCR) was then calculated based on the total FI per egg mass produced per hen.

### 2.3. Egg Quality

At the 50th week of age, 6 eggs per replicate in each treatment group (*n* = 30) were randomly selected to evaluate egg-quality traits. First, eggs were weighed (W), and then they were broken on a flat plate to obtain the internal parts (yolks and albumens). The heights of the albumens (AH) and the yolks (YH) were measured using a tripod micrometer (Baxlo Instrumentos de Medida y Precisión, SL, Barcelona, Spain). The diameters of the albumens (AD) and the yolks (YD) were also measured by a standard caliper (Total Tools, South Melbourne, Australia). The albumen index (AI) and yolk index (YI) were assessed by using the following formulas: AH/AD % and YH/YD %, respectively. The Haugh units (HU) were obtained by the following formula: 100 log (AH—1.7 W^0.37^ + 7.6). The yolk color (YC) score was physically determined using DSM-YC Fan (ORKA Food Technology, LLC, West Bountiful, UT, USA). The shells of the broken eggs were gently washed, air-dried, and then weighed to calculate the shell weight (SW) percent of the egg weight. The shell thickness (ST) was obtained via an eggshell thickness gauge (0–10 mm range, 0.01 mm sensitivity, Baxlo Instrumentos de Medida y Precisión, SL, Barcelona, Spain), based on the average measurements of 3 points on the equatorial regions and the blunt part of the egg. The shell strength (SS) was also measured by applying an assisted system pressure to the egg blunt (Egg Force Reader, ORKA Food Technology LLC, UT, USA).

### 2.4. Metabolites Assay

Immediately after the end of the treatments (50 weeks of age), two blood samples per replicate (*n* = 10) were collected from the brachial vein and transferred into heparinized tubes. The samples were centrifuged at 2000× *g* for 10 min under cooling temperature (4 °C) to separate plasma which was stored at −20 °C for further biochemical analysis. The total protein (TP), albumin (ALB), globulin (GLB), calcium (CA), phosphorus (PP), creatinine (CRT), and uric acid (UA) concentrations in the plasma as well as the activities of aspartate transferase (AST) and alanine transferase (ALT) enzymes were measured using an automated scanning spectrophotometer (CE1010, Cecil Instruments Limited, Cambridge, UK) and commercial colorimetric kits (BioDiagnostic, Giza, Egypt). The full protocol description for each analysis is presented in the Supplementary Materials (File S1).

### 2.5. Hematological Parameters

At the conclusion of the trial (50 weeks of age), blood samples were gathered from the brachial vein of 2 birds per replicate (*n* = 10) and relocated into heparinized tubes to figure out the hematological parameters. Two drops (10 µL) of each sample were diluted with either 490 µL of brilliant cresyl blue stain or 1.990 mL of physiological saline. A drop of each of these two dilutions was mounted on a Bright-Line™ hemocytometer slide (American Optical, Buffalo, NY, USA) to count the total white blood cells (WBC) and red blood cells (RBC), respectively, by using a light microscope at 200× magnification. The hemoglobin (HB) concentration was measured using a colorimetric kit (BioDiagnostic, Giza, Egypt) according to the cyanomethemoglobin method cited in a previous study [38]. The hematocrit (HT) values were determined by reading the packed cell volume on the graduated scale after centrifugation of the blood samples in Wintrobe hematocrit tubes (2000× *g*, 20 min, 4 °C). The mean corpuscular volume (MCV), mean corpuscular hemoglobin (MCH), and MCH concentration (MCHC) were then calculated using the following equations:

MCV [fL] = (HT [%]/RBC [10^6^/µL]) × 10, (1)

MCH [pg/cell] = HB [g/dL]/RBC [10^6^/µL] × 10, (2)

MCHC [g/dL] = HB [g/dL]/HT [%] × 100,(3)

### 2.6. Statistical Analysis

The SPSS software package (version 22.0; IBM Corp., Armonk, NY, USA, 2013) was used to analyze the data. Multivariate tests of the general linear model (GLM) analysis were carried out with respect to the SP inclusion levels into the layer diets as independent variables. Orthogonal polynomial contrasts were performed to test the linear and quadratic trends of the increasing SP levels applied in the present study on all parameters of the productive performance, egg quality, blood metabolites, and hematology. Five replicates of 10 birds per group were statistically analyzed for the productive performance data (*n* = 50), while 6 eggs per replicate were randomly selected in each group for analyzing egg quality data (*n* = 30), and 2 birds per replicate were randomly selected per group to analyze the plasma metabolites and hematological parameters (*n* = 10). Results of the means in addition to the pooled standard error of means (SEM) were presented. A pairwise comparison was used to separate the means considering the level of statistical significance at *p* < 0.05.

## 3. Results

### 3.1. Productive Performance

The effect of SP treatment on the productive performance of laying hens during 40–50 weeks of age is presented in Table 3. The FBW was linearly increased (*p* < 0.05), as the level of the SP increased to 12% into layer diets. The SP treatment linearly and quadratically (*p* < 0.05) increased the EP, EW, and FI, and decreased the FCR. The best values in EP, EW, and FCR were obtained when SP was included into layer diets at 9%, while the best values of FI were observed when using 12% of SP.

### 3.2. Egg Quality

The effect of SP treatment on the egg quality parameters is displayed in Table 4. Results showed that the SP treatment linearly and quadratically increased (*p* < 0.05) the egg AI and HU. Linear increasing trends were obtained in the YI and YC as a response to the increased levels of the dietary SP treatment, and a quadratic effect was also observed in the YC (*p* < 0.05). There was a quadratic increase in the SW and a linear increase in the ST as the dietary SP level increased (*p* < 0.05). In addition, the SP treatment linearly and quadratically (*p* < 0.05) increased the SS, obtaining the highest SS value with 9% SP.

### 3.3. Blood Metabolites 

The effect of SP treatments on the plasma metabolites of laying hens is presented in Table 5. The TP, ALB, and GLB were linearly (*p* < 0.05) increased by the SP treatment, showing the highest values at 9% SP level. In addition, there was a quadratic effect (*p* < 0.05) for the increasing levels of the SP on the TP and GLB. The SP treatments linearly (*p* < 0.05) increased the plasma CA and PP levels, and a quadratic increase was found in the CA as the dietary SP level increased. On the other hand, the SP treatment linearly and quadratically (*p* < 0.05) decreased the plasma AST, ALT, CRT, and UA levels, showing the lowest values in the 9% SP treatment group.

### 3.4. Hematological Parameters

The effect of SP treatments on the hematological analysis of laying hens is shown in Table 6. Results indicated that the WBC was linearly and quadratically (*p* < 0.05) increased as the dietary SP level increased, while the RBC was linearly (*p* < 0.05) increased by increasing the SP level into layer diets. Data also showed that the HB was linearly and quadratically (*p* < 0.05) increased as the dietary SP level increased, while the HT was linearly (*p* < 0.05) increased by increasing the SP level into layer diets. Furthermore, linear and quadratic elevation (*p* < 0.05) in the MCH and MCHC were obtained as the dietary SP levels increased. In contrast, the SP treatment did not affect the MCV of the layers (*p* > 0.05).

## 4. Discussion

In the current study, the possible effect of dietary SP inclusion into layer diets as a complementary source of protein on the hens’ performance was investigated. Therefore, approximately 3.9%, 7.7%, 11.6%, and 15.4% of the SBM as a traditional source of protein in the control basal diet was substituted with 3%, 6%, 9%, and 12% SP, considering the reformulation of the other ingredients to contain the same nutritional composition for all experimental diet groups (Table 2). The dietary SP treatment positively improved the productive performance of laying hens, characterized by increased EP, EW, FI, and decreased FCR of laying hens. The increased FI by the SP treatment may be the direct reason for the linear increase in the FBW and in the other productive parameters of the treated layers [39]. Most of the research that applied the addition of the SP into poultry diets did not indicate any changes in the productive performance of laying hens [13,30,32]. This may be due to either using low levels of the SP (1.0–2.5%) or employing older hens (age > 60 wks) in these studies, compared to the higher SP levels or younger hens used in the present study. Our results indicated that the 9% of the SP treatment group showed the best outcomes regarding the productive performance. In this group, the SBM ingredient was reduced from 275 to 159 g/kg diet, suggesting that 4.7% of SBM of layer diets could be replaced by 1% of SP to optimize the performance of laying hens.

Most of the egg-quality parameters in the present study were enhanced in proportion to the increase in the SP level into the layer diet. Linear and quadratic trends in the quality parameters were evidenced by the increase in the SP levels, especially in the egg YC. In accordance with the presented results, more than 90% of linear correlation between the YC and dietary SP treatment was found in other studies [13,32,33,40]. This correlation is often attributed to the rich SP contents of β-carotene, which is converted into retinol and deposited in egg yolks [41].

According to the nutritional composition of the SBM and SP presented in Table 1, the SP contained higher contents of CP (56.4% versus 44%) and essential amino acids, such as isoleucine (6.6% versus 2.5%), valine (6.6% versus 2.4%), lysine (4.8% versus 2.7%), methionine (2.4% versus 0.7%), and threonine (5.3% versus 1.7%), than that in the SBM. The plasma proteins (TP, ALB, and GLB) were increased by the SP treatment in the current study as well as in previous studies [42,43]. This result could be because of the high contents of SP with protein and at least five essential amino acids [44]. In addition, the increase in plasma proteins may contribute to the increase in egg AI and HU, which were recorded in the SP-treatment groups, through forming a complex with the polysaccharides, polyphenols, and β-ovomucin secreted from the hens’ oviduct [45]. In addition to the finding that the SP used in the current study contains 75% CA more than the SBM (Table 1), it has also been reported that the bioavailability of CA obtained from SP was higher than that obtained from other sources [46]. Our data displayed that SP treatment significantly increased the plasma CA and PP concentration, although the CA contents of the diets were adjusted at the same level. It is an interesting result since the higher level of CA in the plasma of layers fed with SP indicated that a higher absorption of CA occurs in the SP groups compared to the SBM group. Moreover, the increase in CA and PP can explain the high positive correlation obtained in the current study between the SP levels and the egg ST and SS [47].

On the other hand, increasing the SP treatment induced a linear and quadratic decrease in the plasma AST, ALT, CRT, and UA levels, especially when using 9% SP. Similar results were previously reported in laying hens [40], in broiler chickens [12], in other animal species [11,48], and in humans [49]. The abnormality of AST and ALT levels could be a signal to hepatocyte or muscle cell damage [50], while the abnormal levels of CRT and UA could be markers to renal dysfunction [51]. The hepatoprotective properties of SP may also regulate the proteins and amino acids metabolism [52], allowing the release of albumin from the liver to the plasma stream [53]. This finding was supported in the present study by the linear increase in plasma ALB as the dietary SP increased.

The results showed a linear and/or quadratic increase in most of the haematological parameters as the dietary SP treatment increased, and the best values were obtained in the 9% SP group. The beneficial effect of spirulina intake on the blood rejuvenation was confirmed in humans and successfully applied to ameliorate the nutritional anaemia in children and elderly people [54,55]. In poultry species, the SP supplementation at 0.5–1.0% was sufficient to improve some haematological parameters, including RBC, HB, HT, MCV, and MCH, in heat-stressed broilers [12]. An increase in the WBC, RBC, and HB were observed in fish supplemented with 3–10% SP in the diet [56,57]. The increase in WBC, RBC, HB, and, consequently, the other parameters, could be due to the presence of phycocyanin and many important minerals (i.e., iron and calcium) in the spirulina, which might directly modify the blood components [58,59]. Furthermore, the well-known antioxidant properties of SP may explain the relevant increase in blood components and haematological markers in the laying hens [60] and other animals [61,62]. It was reported that such antioxidant compounds can produce a remarkable improvement in the haematological parameters through counteracting the intravascular haemolysis, iron reduction, and erythrocyte deterioration induced by oxidative stress [63].

## 5. Conclusions

The study concludes that SBM of layer diets could be replaced by SP to improve the productive performance, egg quality, blood metabolites, and hematology. There are linear and quadratic effects for increasing the levels of SP inclusion into the layer diets. However, the highest values of most parameters were observed when using 9% SP (90 g/kg of the layer diets). Furthermore, the results suggests that 4.7% of SBM can be replaced by 1% of SP as a promising nutritional approach to optimize the performance of laying hens.

## Figures and Tables

**Table 1:** The chemical analysis of soybean meal (SBM) and *Spirulina platensisis* (SP).

Item	Contents per 100 g SBM *	Contents per 100 g SP
Chemical composition		
Dry matter	90.0 g	94.4 g
Crude protein	44.0 g	56.4 g
Total lipids	0.5 g	7.2 g
Carbohydrate	-	14.2 g
Crude fiber	7.0 g	0.02 g
Total ash	-	7.5 g
Gross energy	-	43.6 MJ
Minerals *		
Calcium	250.0 mg	436.3 mg
Phosphorus	200.0 mg	124.5 mg
Sodium	40.0 mg	220.1 mg
Potassium	1970.0 mg	167.8 mg
Iron	-	11.5 mg
Zinc	-	2.4 mg
Essential amino acids *		
Isoleucine	2.5 g	6.6 g
Leucine	-	8.3 g
Valine	2.4 g	6.6 g
Lysine	2.7 g	4.8 g
Tryptophan	0.6 g	1.1 g
Phenylalanine	-	4.7 g
Methionine	0.7 g	2.4 g
Threonine	1.7 g	5.3 g
Nonessential amino acids *		
Cysteine	0.7 g	1.0 g
Tyrosine	-	4.5 g
Alanine	-	8.5 g
Arginine	3.4 g	7.4 g
Aspartic acid	-	11.5 g
Glutamic acid	-	10.2 g
Glycine	-	5.5 g
Histidine	-	2.5 g
Proline	-	4.3 g
Serine	-	4.9 g

* According to the commercial supplier (not analyzed).

**Table 2:** Composition of the experimental diets ^1^.

Ingredients (g/kg as Fed)	Control	3% SP	6% SP	9% SP	12% SP
Spirulina	0.0	30.0	60.0	90.0	120.0
Soybean meal (44% CP)	275.0	236.4	197.7	159.1	120.5
Yellow corn	566.5	575.1	583.8	592.4	601.0
Wheat bran	10.0	10.0	10.0	10.0	10.0
Soybean oil	30.0	30.0	30.0	30.0	30.0
Bone meal	30.0	30.0	30.0	30.0	30.0
Limestone	80.0	80.0	80.0	80.0	80.0
Salt (NaCl)	4.0	4.0	4.0	4.0	4.0
Premix ^2^	3.0	3.0	3.0	3.0	3.0
DL-Methionine	1.5	1.5	1.5	1.5	1.5
Calculated nutrients					
Metabolizable energy (MJ/kg)	12.6	12.6	12.6	12.6	12.6
Calcium (g/kg)	40.2	40.2	40.2	40.2	40.2
Available phosphorus (g/kg)	5.2	5.2	5.2	5.2	5.2
Determined nutrients					
Crude protein (g/kg)	167.5	170.0	170.0	174.5	174.7
Crude fat (g/kg)	66.0	64.5	63.8	62.1	61.5
Crude fiber (g/kg)	47.0	46.5	46.5	45.8	45.5

^1^ Experimental diets: supplemented with 3% spirulina (3% SP), 6% spirulina (6% SP), 9% spirulina (9% SP), 12% spirulina (12% SP), or without spirulina (Control). ^2^ Premix (content per kg of the experimental diet): 8000 IU vitamin A; 1500 IU vitamin D; 4 mg riboflavin; 10 µg cobalamin; 15 mg vitamin E; 2 mg vitamin K; 500 mg choline; 25 mg niacin; 60 mg manganese; 50 mg zinc.

**Table 3:** Effect of dietary *Spirulina platensis* on the productive performance of laying hens.

Parameters ^1^	Dietary *Spirulina platensis* (SP) Groups					SEM	*p*-Value		
	Control	3% SP	6% SP	9% SP	12% SP		Treatment	Linear	Quadratic
IBW, g	1550.0	1549.7	1549.8	1550.8	1550.1	2.26	0.998	0.863	0.981
FBW, g	1597.4 ^b^	1597.0 ^b^	1599.0 ^ab^	1600.8 ^ab^	1604.7 ^a^	2.42	0.153	0.016	0.354
Hen-day EP, %	89.2 ^c^	88.9 ^c^	91.4 ^b^	92.9 ^a^	91.8 ^b^	0.19	<0.001	<0.001	<0.001
EW, g	61.2 ^d^	61.2 ^d^	62.1 ^b^	62.9 ^a^	61.7 ^c^	0.09	<0.001	<0.001	<0.001
FI, g/d	99.5 ^c^	99.5 ^c^	100.9 ^b^	101.4 ^b^	104.4 ^a^	0.19	<0.001	<0.001	<0.001
FCR	1.82 ^b^	1.83 ^ab^	1.78 ^c^	1.74 ^d^	1.84 ^a^	0.006	<0.001	0.011	<0.001

^1^ Parameters: IBW, initial body weight at the 40th week of age; FBW, final body weight at the 50th week of age; EP, egg production; EW, egg weight; FI, feed intake; FCR, feed conversion ratio. Data are presented as means for five replicates of 10 hens each (*n* = 50 per group). Means within the same row with different superscripts are significantly different (*p* < 0.05). SEM, pooled standard error of means.

**Table 4:** Effect of dietary *Spirulina platensis* on the egg quality parameters of laying hens.

Parameters ^1^	Dietary *Spirulina platensis* (SP) Groups					SEM	*p*-Value		
	Control	3% SP	6% SP	9% SP	12% SP		Treatment	Linear	Quadratic
AI, %	9.87 ^b^	10.47 ^ab^	10.93 ^a^	10.70 ^a^	10.67 ^a^	0.246	0.034	0.020	0.037
HU	81.92 ^b^	85.44 ^a^	87.80 ^a^	87.71 ^a^	85.70 ^a^	0.839	<0.001	<0.001	<0.001
YI, %	40.18 ^b^	40.95 ^ab^	41.19 ^ab^	42.60 ^a^	41.83 ^ab^	0.702	0.153	0.028	0.464
YC	7.90 ^e^	8.50 ^d^	9.87 ^c^	11.20 ^b^	12.67 ^a^	0.124	<0.001	<0.001	<0.001
SW, %	8.71 ^b^	8.89 ^ab^	9.02 ^ab^	9.18 ^a^	8.80 ^ab^	0.141	0.152	0.288	0.042
ST, mm	0.32^c^	0.33 ^c^	0.37 ^b^	0.42 ^a^	0.42 ^a^	0.008	<0.001	<0.001	0.439
SS, kg/cm^2^	3.84 ^c^	3.95 ^c^	4.43 ^b^	4.72 ^a^	4.49 ^b^	0.070	<0.001	<0.001	0.001

^1^ Parameters: AI, albumen index; HU, Haugh units; YI, yolk index; YC, yolk color; SW, shell ratio; ST, shell thickness; SS, shell strength. Data are presented as means for five replicates of six eggs each (*n* = 30 per group). Means within the same row with different superscripts are significantly different (*p* < 0.05). SEM, pooled standard error of means.

**Table 5:** Effect of dietary *Spirulina platensis* on the blood biochemicals of laying hens.

Parameters ^1^	Dietary *Spirulina platensis* (SP) Groups					SEM	*p*-Value		
	Control	3% SP	6% SP	9% SP	12% SP		Treatment	Linear	Quadratic
TP, g/dL	4.62 ^e^	5.27 ^d^	6.16 ^c^	7.68 ^a^	6.81 ^b^	0.138	<0.001	<0.001	<0.001
ALB, g/dL	2.97 ^c^	3.42 ^b^	3.52 ^b^	4.32 ^a^	4.09 ^a^	0.106	<0.001	<0.001	0.096
GLB, g/dL	1.65 ^c^	1.85 ^c^	2.64 ^b^	3.36 ^a^	2.72 ^b^	0.122	<0.001	<0.001	<0.001
CA, mg/dL	25.58 ^d^	28.59 ^c^	31.21 ^b^	32.56 ^a^	31.76 ^ab^	0.384	<0.001	<0.001	<0.001
PP, mg/dL	6.40 ^b^	6.35 ^b^	6.50 ^b^	7.08 ^a^	6.90 ^a^	0.084	<0.001	<0.001	0.608
AST, U/mL	63.70 ^a^	62.15 ^a^	58.52 ^b^	57.19 ^b^	57.55 ^b^	0.755	<0.001	<0.001	0.035
ALT, U/mL	36.86 ^a^	32.68 ^b^	29.57 ^c^	22.17 ^e^	25.31 ^d^	0.660	<0.001	<0.001	<0.001
CRT, mg/dL	0.28 ^a^	0.27 ^ab^	0.26 ^bc^	0.25 ^d^	0.26 ^cd^	0.005	<0.001	<0.001	0.037
UA, mg/dL	5.71 ^a^	5.43 ^b^	5.24 ^c^	4.94 ^d^	5.13 ^cd^	0.068	<0.001	<0.001	0.002

^1^ Parameters: TP, total protein; ALB, albumin; GLB, globulin; CA, calcium; PP, phosphorus; AST, aspartate transferase; ALT, alanine transferase; CRT, creatinine; UA, uric acid. Data are presented as means for five replicates of two hens each (*n* = 10 per group). Means within the same row with different superscripts are significantly different (*p* < 0.05). SEM, pooled standard error of means.

**Table 6:** Effect of dietary *Spirulina platensis* on the blood hematology of laying hens.

Parameters ^1^	Dietary *Spirulina platensis* (SP) Groups					SEM	*p*-Value		
	Control	3% SP	6% SP	9% SP	12% SP		Treatment	Linear	Quadratic
WBC, 10^3^/µL	36.18 ^e^	42.73 ^d^	51.60 ^c^	66.78 ^a^	58.08 ^b^	1.375	<0.001	<0.001	<0.001
RBC, 10^6^/µL	2.24 ^c^	2.25 ^c^	2.29 ^bc^	2.39 ^a^	2.34 ^ab^	0.022	<0.001	<0.001	0.454
HB, g/dL	10.85 ^b^	10.68 ^b^	12.90 ^a^	13.36 ^a^	11.61 ^b^	0.329	<0.001	<0.001	<0.001
HT, %	30.58 ^c^	30.49 ^c^	31.51 ^bc^	32.86 ^a^	31.76 ^b^	0.380	<0.001	<0.001	0.240
MCV, fL	136.36	135.89	137.59	137.63	136.16	2.328	0.973	0.855	0.677
MCH, pg/cell	48.38 ^b^	47.60 ^b^	56.30 ^a^	55.97 ^a^	49.80 ^b^	1.548	<0.001	0.027	0.001
MCHC, g/dL	35.49 ^b^	35.11 ^b^	41.02 ^a^	40.64 ^a^	36.61 ^b^	1.109	<0.001	0.032	0.002

^1^ Parameters: WBC, white blood cells; RBC, red blood cells; HB, hemoglobin; HT, hematocrit; MCV, mean corpuscular volume; MCH, mean corpuscular hemoglobin; MCHC, mean corpuscular hemoglobin concentration. Data are presented as means for five replicates of two hens each (*n* = 10 per group). Means within the same row with different superscripts are significantly different (*p* < 0.05). SEM, pooled standard error of means.

## Data Availability

Not applicable.

## Supplementary Materials

The following supporting information can be downloaded at: https://www.mdpi.com/article/10.3390/ani12202816/s1, File S1: The protocol description for metabolites assay.
