# Supplementary material for: Effect of Dietary Blue-Green Microalgae Inclusion as a Replacement to Soybean Meal on Laying Hens’ Performance, Egg Quality, Plasma Metabolites, and Hematology
Source: Animals (Basel). 2022 Oct 18;12(20):2816. doi: 10.3390/ani12202816 (PMC9597824; doi:10.3390/ani12202816)
Supplement: Supplementary file 1 [file animals-12-02816-s001.zip › File S1/albumin(2).pdf]

# ALBUMIN

**Colorimetric Method** **100 Tests**

## PRINCIPLE :

**Formation of an albumin/bromcresol – green complex at pH 3.8 and photometric measurement of the absorbnance.**

## SAMPLE :

**Serum, or plasma the samples can be stored up to two days at +4˚C.**

## NORMAL VALUES :

**Children:** 3.8 – 4.2 g/dl
**Adults:** 3.5 – 5.5 g/dl

## REAGENTS :

| | | |
|---|---|---|
| 1- | Standard<br>Albumin | 4 g/dl |
| 2- | Color Reagent<br>Citrate buffer, pH 3.8<br>Bromcresol green<br>Detergent<br>Preservative | 50 mmol/l<br>0.12 mmol/l |

## STABILITY :

**The reagents are Stable up to the expiry date specified when stored at +4 to +8°C .**

## PROCEDURE:

| | Blank (ml) | Standard (ml) | Sample (ml) |
|---|---|---|---|
| Standard | - | 0.01 | - |
| Sample | - | - | 0.01 |
| Reagent 2 | 2.0 | 2.0 | 2.0 |

**Mix well, then measure after 5 min. the absorbances of sample ($A_{sample}$) and the standard ($A_{standard}$) against reagent blank at 630 nm (620 – 640 nm ).**

**If albumin concentration exceeds 6 g/dl dilute the sample and repeat the assay. Multiply by the dilution factor.**

## CALCULATION :

**Albumin Concentration (g/dl)**

$$= \frac{A_{Sample}}{A_{Standard}} \times 4$$

## REFERENCE :

**Doumas B.T et al., (1971): Clin. Chim. Acta 31-87.**

## QUALITY CONTROL:

**For accuracy and reproducibility control:**
**Assayed Multi-Sera Normal and Elevated.**

BIODIAGNOSTIC
DIAGNOSTIC AND RESEARCH REAGENTS

# ALBUMIN

**Colorimetric Method**

**+4 to +8ºC** **100 Tests**

**In vitro diagnostic use**

**CAT. No.** **AB 10 10**

# REAGENTS

| | | |
|---|---|---|
| **R1** | **Standard** | **2 ml** |
| **R2** | **Color Reagent** | **2 x 100 ml** |

# CONTACTS

Tele: 02-33385184
Mobil: 0109 – 349 20 77
Fax : 02-33385184 (102)
e.maile : info@bio-diagnostic.com
Website: www.bio-diagnostic.com
Adress: 29 Tahreer St., Dokki, Giza, Egypt
