# Supplementary material for: Effect of Dietary Blue-Green Microalgae Inclusion as a Replacement to Soybean Meal on Laying Hens’ Performance, Egg Quality, Plasma Metabolites, and Hematology
Source: Animals (Basel). 2022 Oct 18;12(20):2816. doi: 10.3390/ani12202816 (PMC9597824; doi:10.3390/ani12202816)
Supplement: Supplementary file 1 [file animals-12-02816-s001.zip › File S1/calcium.pdf]

CALCIUM

Colorimetric Method **50 Tests**

PRINCIPLE :

Calcium ion produces with methylthymol blue, in an alkaline medium, a blue color the intensity of which is in proportion to the calcium concentration. The presence of hydroxy 8-quinoline eliminate the interference due to the magnesium ions.

SAMPLE :

Serum. heparinized plasma or urine. Dilute urine 1 + 3 multiply result by 4 .

NORMAL VALUES :

**Serum Adults:**		**8.1 – 10.4 mg / dL**
		**(2.02 –2.6 mmol/L)**
**Children**	**:**	**10 – 11.5 mg / dL**
**Urine**	**:**	**140 - 350 mg / 24 hrs**

REAGENTS :

**1.**	**Standard**	10 mg / dL (2.5 mmol/L)
**2.**	**Chromogen :**	
	Methylthymol blue	0.2 mmol /L
	Hydroxy 8-quinoline	140 mmol /L
	Hydrochloric acid	200 mmol/L
**3.**	**Buffer :**	
	Ethanolamine	6 mol / L

STABILITY :

All reagents are stable up to the expiry date specified when stored at +15 to +25°C .

PROCEDURE :

Rinse test tubes before use with working reagent if necessary.

Working reagent : Mix one volume of reagent 2 with one volume of reagent 3 befor use .

Pipette into test tubes (FREE OF CALCIUM)

	Blank ml	Standard ml	Sample ml
**Sample**	-	-	0.02
**Standard (R1)**	-	0.02	-
**Working reagent**	1.0	1.0	1.0

Mix well, wait 5 min. at room temperature . Read the absorbances of the sample (A_{Sample}) and the standard ($A_{Standard}$) against blank, at 585 nm. (575 - 590) . The color is stable for at least 60 min. Linearity up to 20 mg / dL .

CALCULATION :

Calcium Concentration

$$= \frac{A_{Sample}}{A_{Standard}} \times \text{Standard Conc.}$$

QUALITY CONTROL :

**For accuracy and reproducibility control:-
Assayed Multi – Sera, Normal and Elevated**

REFERENCE :

Gindler M. King J.D. (1972) : Am. J. Clin. Path. 58, 376.

BIODIAGNOSTIC
DIAGNOSTIC AND RESEARCH REAGENTS

CALCIUM

Colorimetric Method

+15 to +25 ºC **50 Tests**

In vitro diagnostic use

**CAT. NO.**	**CA 12 10**

REAGENTS

**R1**	**Standard**	**2ml**
**R2**	**Chro mogen**	**25ml**
**R3**	**Buffer**	**25ml**

CONTACTS

Tele: 02-33385184
Mobil: 0109 – 349 20 77
Fax : 02-33385184 (102)
e.maile : info@bio-diagnostic.com
Website: www.bio-diagnostic.com
Adress: 29 Tahreer St., Dokki, Giza, Egypt
