# Supplementary material for: Effect of Dietary Blue-Green Microalgae Inclusion as a Replacement to Soybean Meal on Laying Hens’ Performance, Egg Quality, Plasma Metabolites, and Hematology
Source: Animals (Basel). 2022 Oct 18;12(20):2816. doi: 10.3390/ani12202816 (PMC9597824; doi:10.3390/ani12202816)
Supplement: Supplementary file 1 [file animals-12-02816-s001.zip › File S1/creatinine_end(1).pdf]

CREATININE

Colorimetric Method (End Point)   100 Tests

PRINCIPLE :

Creatinine forms a colored complex with picrate in an alkaline medium .

.

SAMPLES :

Serum, heparinized plasma and urine.
Dilute fresh urine 1 + 49 with dist. water .

NORMAL VALUES :

Serum:Men: 0.6 – 1.1 mg / dl (53 – 97 µmol/L)
Women:0.5 – 0.9 mg / dl (44 – 80 µmol/L)
Urine : 1.0 – 2.0 g / 24 hrs.
Creatinine clearance : Men:98 – 156 ml / min.
Women: 95 – 160 ml / min.

REAGENTS :

**1.**	Standard	2 mg / dL (177 µmol/L)
**2.**	Picric Acid	20 mmol / L
**3.**	Sodium hydroxide	1.2 mmol / L
	Additional reagent: (available on request) Trichloroacetic acid (TCA) 1.2 mol / L	

STABILITY :

The reagents are stable up to the expiry date specified when stored at +15 to +25 °C .

DEPROTEINIZATION :

Pipette into centrifuge tube :	
Trichloroacetic acid	0.5 ml
Serum or heparinized plasma	0.5 ml

Mix well. Wait for 5 min. Centrifuge for 10 min. at 3000 rpm , then carefully pour the clear supernatant into dry test tube. The supernatant can be stored to seven days at +4 °C .

PROCEDURE :

Working reagent
Mix equal volumes of reagents 2 and 3 immediately before the assay .

	Blank mL	Standard mL	Serum mL	Urine mL
Dis. H_2O	0.25	-	-	-
Reagent 1	-	0.25	-	-
TCA	0.25	0.25	-	0.25
Serum supernate	-	-	0.5	-
Urine diluted	-	-	-	0.25
Working Reagent	0.5	0.5	0.5	0.5

Mix. Incubate 5 min. at 37°C. Measure the absorbances of sample (A_{Sample}) and standard ($A_{Standard}$) against the blank at 520 nm.(500 – 550 nm).Linearity up to10 mg / dl in serum or plasma and 300 mg /dl in diluted urine .

CALCULATION :

Creatinine in serum or plasma (mg/ dl) = $\frac{A_{Sample}}{A_{Standard}} \times 2$

Creatinine in urine (mg / dl) = $\frac{A_{Sample}}{A_{Standard}} \times 100$

Creatinine Clearance (ml / min.)

$$= \frac{\text{mg creatinine / dl urine x ml urine / 24 hrs}}{\text{mg creatinine / dl serum x 1440}}$$

QUALITY CONTROL :

For Accuracy and reproducibility control:-
Assayed Multi – Sera, Normal and Elevated .

REFERENCE :

Schirmeister ., J. et al . (1964) : Dtsch.med Wschr 89 : 1940

BIODIAGNOSTIC
DIAGNOSTIC AND RESEARCH REAGENTS

CREATININE

Colorimetric Method
(End Point)
+15 to +25ºC 100 Tests
In vitro diagnostic use

CAT. No. CR 12 50

Biodiagnostic BD

REAGENTS

R1 Standard	25 ml
R2 Picric acid	25 ml
R3 Sodium hydroxide	25 ml

CONTACTS

Tele: 02-33385184
Mobil: 0109 – 349 20 77
Fax : 02-33385184 (102)
e.maile : info@bio-diagnostic.com
Website: www.bio-diagnostic.com
Adress: 29 Tahreer St., Dokki, Giza, Egypt
