# Supplementary material for: Effect of Dietary Blue-Green Microalgae Inclusion as a Replacement to Soybean Meal on Laying Hens’ Performance, Egg Quality, Plasma Metabolites, and Hematology
Source: Animals (Basel). 2022 Oct 18;12(20):2816. doi: 10.3390/ani12202816 (PMC9597824; doi:10.3390/ani12202816)
Supplement: Supplementary file 1 [file animals-12-02816-s001.zip › File S1/got_+_gpt.pdf]

GOT (AST) Glutamic – Oxaloacetic Transaminase
GPT (ALT) Glutamic – Pyruvic Transaminase

Colorimetric Method — **GOT 100 Tests** / **GPT 100 Tests**

PRINCIPLE :

Colorimetric determination of GOT (AST) or GPT (ALT) activities according to the following reactions :

$$\text{Aspartate} + \alpha\text{-Ketoglutarate} \overset{GOT}{\rightleftarrows} \text{oxaloacetate} + \text{glutamate}$$

$$\text{Alanine} + \alpha\text{-ketoglutarate} \overset{GPT}{\rightleftarrows} \text{pyruvate} + \text{glutamate}$$

The keto acids pyruvate or oxaloacetate formed is measured in its derivative form, 2,4- dinitrophenylhydrazone.

SAMPLES :

Serum. Hemolysis will interfere. Use fresh sample.

NORMAL VALUES :

GOT: up to 40 Units / ml GPT : up to 45 Units / ml

REAGENTS :

**1.**	**GOT Buffer substrate:**<br>Phosphate buffer pH 7.5<br>Aspartate<br>α-Ketoglutarate	 100 mmol/L<br>100 mmol/L<br>2 mmol/L
**2.**	**GPT Buffer substrate :**<br>Phosphate buffer pH 7.5<br>Alanine<br>α-Ketoglutarate	 100 mmol/L<br>200 mmol/L<br>2 mmol/L
**3.**	**Color Reagent :**<br>2,4 dinitrophenylhydrazine	 1 mmol/L
**4.**	**Standard pyruvate**	2 mmol/L
	**Additional reagent:** (available on request)<br>Sodium hydroxide	 0.4 N

STABILITY :

The reagents are stable up to the expiry date specified when stored at +4 to +8 °C .

PROCEDURE :

Set up the following tubes for each sample:

	GOT (ml)	GPT (ml)
Reagent 1	0.5	—
Reagent 2	—	0.5
Incubate for 5 min. at 37°C		
Serum	0.1	0.1
Mix and incubate at 37°C for :	exactly 60 min	exactly 30 min
Reagent 3	0.5	0.5
Mix. Let stand for 20 min. at room temperature.		
NaOH 0.4 N	5.0	5.0

Mix. Wait 5 min. Measure the absorbances at 505 nm (490 – 520 nm) against d. Water using cuvettes 1 cm light path. The color is stable for one hour. Linearity for GOT up to 150 units/ml and for GPT up to 120 units /ml .

CALCULATION :

Calculate the number of units / ml of GOT and GPT of sample using the standard curve .

QUALITY CONTROL :

For Accuracy and reproducibility control:-
Assayed Multi – Sera, Normal and Elevated.

Standard Curve

Pipette into test tubes (ml) :

Tube No.	1	2	3	4	5
D. Water	0.1	0.1	0.1	0.1	0.1
Reagent (1or 2)	0.5	0.45	0.4	0.35	0.30
Pyruvate (R4)	-	0.05	0.1	0.15	0.2
Reagent (3)	0.5	0.5	0.5	0.5	0.5
Mix. Let stand for 20 min. at room temperature					
NaOH 0.4 N	5	5	5	5	5
Mix. Wait 5 min. Measure as for test at 505 nm					
GOT units/ ml	0	22	55	95	150
GPT units/ ml	0	25	50	83	126

Plot the standard curve :

- <u>Abscissa :</u> number of units / ml

- <u>Ordinate :</u> OD (505 nm)

REFERENCE :

Reitman, A. and Frankel, S. (1957) : Amer J. Clin. Path ., 28 : 56.

BIODIAGNOSTIC

DIAGNOSTIC AND RESEARCH REAGENTS

GOT (AST)

Glutamic – Oxaloacetic Transaminase

GPT (ALT)

Glutamic – Pyruvic Transaminase

Colorimetric Method

+4 to +8˚C

GOT 100 Tests

GPT 100 Tests

In vitro diagnostic use

CAT. NO. AT 10 34 (45)

REAGENTS

R1	GOT- Buffer Substrate	50 ml
R2	GPT- Buffer Substrate	50 ml
R3	Color Reagent	2 x 50 ml
R4	Standard Pyruvate	3 ml

CONTACTS

Tele: 02-33385184
Mobil: 0109 – 349 20 77
Fax : 02-33385184 (102)
e.maile : info@bio-diagnostic.com
Website: www.bio-diagnostic.com
Adress: 29 Tahreer St., Dokki, Giza, Egypt
