# Supplementary material for: Effect of Dietary Blue-Green Microalgae Inclusion as a Replacement to Soybean Meal on Laying Hens’ Performance, Egg Quality, Plasma Metabolites, and Hematology
Source: Animals (Basel). 2022 Oct 18;12(20):2816. doi: 10.3390/ani12202816 (PMC9597824; doi:10.3390/ani12202816)
Supplement: Supplementary file 1 [file animals-12-02816-s001.zip › File S1/phosphorus.pdf]

PHOSPHOROS

Colorimetric Method **50 Tests**

PRINCIPLE :

Inorganic phosphorus present in serum as phosphate forms a phosphomolybdate complex with molybdic acid. The complex is reduced by stannous chloride to a blue color which can be measured colorimetrically. Formic acid used as protein solubilizer and glycerol as stabilizer for assay system .

SAMPLES :

Serum or plasma . Hemolysis will interfere.
Urine (dil.10 times)

NORMAL VALUES :

Plasma or serum
Adult : 2.5 – 5 mg / dL (0.80 – 1.61 mmol/L).
Children : 4 – 7 mg / dL (1.28 – 2.25 mmol/L).
Urine :0.35 – 1.0 g (11.2 – 32 mmoL) / day .

REAGENTS :

1.	**Standard**	4<br>1.288	mg/dl<br>mmol/L
2.	**Color Reagent**<br>Formic acid<br>Glycerol<br>Ammonium molybdate	 2<br>0.6<br>0.32	 mol/L<br>mol/L<br>mmol/L
3.	**Reducing agent**<br>Stannous chloride	 2	 mmol/L

STABILITY :

The reagents are stable up to the expiry date specified when stored at +15 to +25 °C .

PROCEDURE :

Dilute reagent 3, 100 times immediately befor use (0.1 ml + 10.0 ml dist water) . Pipette into test tubes (PHOSPHORUS – FREE) :

	Blank (ml)	Standard (ml)	Sample (ml)
Standard	-	0.025	-
Sample	-	-	0.025
Reagent 2	1.0	1.0	1.0
Mix, then add :			
Reagent 3 (diluted)	0.1	0.1	0.1

Mix. let stand for 10 min. at 37°C . Read the absorbances of sample (A_{Sample}) and standard ($A_{Standard}$) against the blank at 640 nm. (630 – 650. nm.) . Color stable for one hour . Linearity up to 15 mg / dL .

CALCULATION :

Phosphorus Concentration in Serum or plasma

$$= \frac{A_{Sample}}{A_{Standard}} \times \text{Standard Conc.}$$

N.B. in case of urine multiply result by 10 (dil.factor)

REFERENCE :

El-Merzabani.M.M.; El- Aaser. A.A. and Zakhary, N.I. (1977) . J. Clin. Chem. Clin. Biochem. 15 : 715 – 718 .

QUALITY CONTROL :

For accuracy and reproducibility control:-
Assayed Multi – Sera, Normal and Elevated

BIODIAGNOSTIC
DIAGNOSTIC AND RESEARCH REAGENTS

PHOSPHORUS

Colorimetric Method

+ 15 to +25 ºC **50 Tests**

In vitro diagnostic use

CAT. No. PH 17 10

REAGENTS

R1	Standard	2.5 ml
R2	Color Reagent	50 ml
R3	Reducing agent	2.5 ml

CONTACTS

Tele: 02-33385184
Mobil: 0109 – 349 20 77
Fax : 02-33385184 (102)
e.maile : info@bio-diagnostic.com
Website: www.bio-diagnostic.com
Adress: 29 Tahreer St., Dokki, Giza, Egypt
