# Supplementary material for: Effect of Dietary Blue-Green Microalgae Inclusion as a Replacement to Soybean Meal on Laying Hens’ Performance, Egg Quality, Plasma Metabolites, and Hematology
Source: Animals (Basel). 2022 Oct 18;12(20):2816. doi: 10.3390/ani12202816 (PMC9597824; doi:10.3390/ani12202816)
Supplement: Supplementary file 1 [file animals-12-02816-s001.zip › File S1/total_protein.pdf]

# PROTEIN - Biuret Method

**Colorimetric Method** **200 Tests**

## PRINCIPLE :

In the presence of an alkaline cupric sulfate, the protein produces a violet color, the intensity of which is proportional to their concetration .

## SAMPLE :

Serum, or plasma, Hemolysis will interfere with the test

## NORMAL VALUES :

**Adults :** 6.5 – 8.7 g /dL .
**Children :** 5.5 – 8.5 g / dL .
**Neonates :** 5.3 – 8.9 g / dL .

## REAGENTS :

| | | |
|---|---|---|
| 1- | **Standard Abumin** | 5 g / dL |
| 2- | **Biuret Reagent:** | |
| | Cupric sulfate | 6 mmol / L |
| | Sodium potassium tartrate | 21 mmol / L |
| | Sodium hydroxide | 750 mmol / L |
| | Potassium iodide | 6 mmol / L |

## STABILITY :

The reagents are stable up to the expiry date specified when stored at +4 to +8 °C away from light .

## PROCEDURE :

| | Blank ( ml ) | Standard ( ml ) | Sample ( ml ) |
|---|---|---|---|
| Standard | - | 0.025 | - |
| Sample | - | - | 0.025 |
| Reagent 2 | 1.0 | 1.0 | 1.0 |

Mix well, incubate for 10 min. at 37°C. Read the absorbances of the sample ($A_{Sample}$ ) and standard ($A_{standard}$ ) against reagent blank at 550 nm. ( 520 - 570 nm) . Color stable for one hour. Linearity up to 10 g / dL .

## CALCULATION :

$$\text{Protein Concentration (g /dL)} = \frac{A_{Sample}}{A_{Standard}} \times 5$$

## REFERENCE :

Gornal A.C; Bardawill C.J.and David M.M. (1949 ) : J. Biol. Chem. 177 : 751

## QUALITY CONTROL :

For accuracy and reproducibility control:-
Assayed Multi – Sera, Normal and Elevated .

BIODIAGNOSTIC
DIAGNOSTIC AND RESEARCH REAGENTS

# PROTEIN Biuret Method

**Colorimetric Method**

**+4 to +8ºC** **200 Tests**

**In vitro diagnostic use**

CAT. NO. TP 20 20

# REAGENTS

| | | |
|---|---|---|
| **R1** | **Standard** | **5 ml** |
| **R2** | **Biuret reagent** | **2 x 100 ml** |

# CONTACTS


Tele: 02-33385184
Mobil: 0109 – 349 20 77
Fax : 02-33385184 (102)
e.maile : info@bio-diagnostic.com
Website: www.bio-diagnostic.com
Adress: 29 Tahreer St., Dokki, Giza, Egypt
