# Supplementary material for: Effect of Dietary Blue-Green Microalgae Inclusion as a Replacement to Soybean Meal on Laying Hens’ Performance, Egg Quality, Plasma Metabolites, and Hematology
Source: Animals (Basel). 2022 Oct 18;12(20):2816. doi: 10.3390/ani12202816 (PMC9597824; doi:10.3390/ani12202816)
Supplement: Supplementary file 1 [file animals-12-02816-s001.zip › File S1/uric_acid.pdf]

URIC ACID

Enzymatic Colorimetric Method **50 Tests**

PRINCIPLE :

Uric acid + $2H_2O$ + O_2 $\xrightarrow{\text{Uricase}}$ Allantoin + CO_2 + H_2O_2

H_2O_2 + 3.5, Dichloro-2- hydroxybenzensulphonate +

4-Amino antipyrine $\xrightarrow{\text{Peroxidase}}$ H_2O + HCL + Colored quinoneimine

SAMPLE :

Serum . plasma or urine
Hemolysis interferes with the test. Heat the urine for 10 min. at 60°C. Centrifuge if the urine is turbid .
Dilute urine 10 times before the assay (1+9 ml/d.H_2O).

NORMAL VALUES :

Serum: Men 3 – 7 mg / dl (178.5 – 416.5 µmol/L).
Women 2 – 6 mg / dl (119 – 357 µmol/L).
Urine: 250 – 750 mg / 24 hrs (1.5 – 4.5 mmol/24 hrs).

REAGENTS :

1.	**Standard**	6 mg / dL (395 µmol/L)
2.	**Chromogen - Buffer**	
	**Tris buffer**	50 mmol / L
	3,5, Dichloro – 2 – hydroxybenzen sulphonate	5.0 mmol / L
	Surface – active agent .	
3.	**Enzymes :**	
	Uricase	> 500 IU / L
	Peroxidase	> 2000 U / L
	4 -Aminoantipyrine	0.20 mmol /L
	Stabilizer and preservative	

STABILITY :

The reagents are stable up to the expiry date specified when stored at +4 to +8 °C away from light .

PROCEDURE :

Working reagent : Mix equal volumes of reagent 2 and 3 immediately befor the assay .

	Blank ml	Standard ml	Sample ml
Standard	-	0.05	-
Sample	-	-	0.05
Working reagent	1.0	1.0	1.0

Mix, incubate for 10 min. at 37°C. Read the absorbances of the sample (A_{Sample}) and the standard ($A_{Standard}$) against blank, at 510 nm. (490 - 550) . Color is stable for 30 min. Linearity up to 30 mg / dL .

CALCULATION :

Uric Acid in serum

$$= \frac{A_{Sample}}{A_{Standard}} \text{ X Standard Conc.}$$

Uric acid in urine (mg / dl) $= \frac{A_{Sample}}{A_{Standard}}$ **x 100**

REFERENCE :

Barham, D., Trinder, P ., (1972) : Analyst, 97 , 142 .

QUALITY CONTROL :

For accuracy and reproducibility control:-
Assayed Multi – Sera, Normal and Elevated

BIODIAGNOSTIC
DIAGNOSTIC AND RESEARCH REAGENTS

Standard Curve of Uric Acid

mg/dL	O.D 510 nm
2.50	0.12
5.00	0.25
10.00	0.51
15.00	0.77
20.00	1.06
25.00	1.34
30.00	1.61

URIC ACID

Enzymatic Colorimetric Method
+4 to +8ºC **50 Tests**
In vitro diagnostic use

CAT. NO. **UA 21 20**

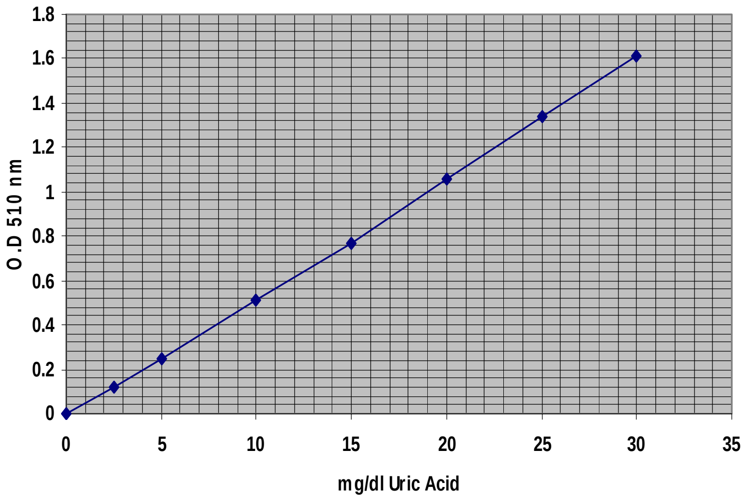

REAGENTS

R1	Standard	2.5 ml
R2	Chromogen- Buffer	25 ml
R3	Enzymes	25 ml

CONTACTS

Tele: 02-33385184
Mobil: 0109 – 349 20 77
Fax : 02-33385184 (102)
e.maile : info@bio-diagnostic.com
Website: www.bio-diagnostic.com
Adress: 29 Tahreer St., Dokki, Giza, Egypt
